# Impact of Chemotherapy on Motor–Cognitive Dual-Task Performance in Strength and Mobility Tests

**DOI:** 10.3390/healthcare13202649

**Published:** 2025-10-21

**Authors:** Almudena Martínez-Sánchez, Candela Guerrero-Torrico, Francisco Javier Dominguez-Muñoz, Narcis Gusi, Santos Villafaina

**Affiliations:** 1 Departamento de Didáctica de la Expresión Musical, Plástica y Corporal, Facultad de Ciencias del Deporte, Grupo de Investigación en Actividad Física, Calidad de Vida y Salud (AFYCAV), Universidad de Extremadura, Avenida de la Universidad s/n, 10003 Cáceres, Spain; almudenams@unex.es (A.M.-S.); cguerrerfq@alumnos.unex.es (C.G.-T.); ngusi@unex.es (N.G.); 2 Instituto de Investigación e Innovación en el Deporte (INIDE), 10003 Cáceres, Spain

**Keywords:** cancer, physical condition, exercise, balance, fitness, cognitive deficit

## Abstract

**Background/Objectives**: Chemotherapy often leads to persistent physical and cognitive impairments, and while the dual-task paradigm is a sensitive tool for detecting such deficits, its application to functional strength in oncology remains largely unexplored. **Methods**: This cross-sectional study, while not designed to establish causality, included 44 participants including cancer patients (11 undergoing chemotherapy and 15 post-treatment survivors) and healthy controls. Functional fitness was assessed with the Senior Fitness Test battery under single- and dual-task conditions. The dual-task condition incorporated a cognitive subtraction task, and the dual-task cost was calculated. Group comparisons were analyzed using *t*-tests and ANOVA with effect sizes reported (*p* < 0.05). **Results**: Within-group comparisons showed that the cancer group exhibited reduced performance from the single- to the dual-task condition, with significant declines in strength and mobility (Chair Stand Test, *p* = 0.011; Timed Up and Go, *p* < 0.001). Greater dual-task costs were observed in the Arm Curl Test for cognitive (*p* = 0.005) and success (*p* = 0.004) outcomes. **Conclusions**: Dual-task testing revealed greater impairments in cancer patients and survivors, supporting the impact of chemotherapy-related cognitive impairment and highlighting the need for integrated motor–cognitive rehabilitation strategies. Further research is warranted in this field, employing larger sample sizes and stratified analyses by specific cancer types, and including a spectrum of complexity in the cognitive task to characterize the effects of cancer and its treatment on motor–cognitive integration.

## 1. Introduction

Cancer remains a major global health challenge, accounting for nearly 20 million new cases and 9.7 million deaths in 2022, representing about one in six deaths worldwide [1]. Despite the alarming global burden of cancer, advances in early detection and treatment have significantly improved survival rates over the past decades. Since the 2000s, 5-year survival rates have reached 30–90% in some types of cancer [2]. Targeted therapies, immunotherapy, and optimized multimodal treatment protocols have contributed to these gains, transforming certain cancers from acutely fatal conditions into chronic, manageable diseases [3,4].

However, both cancer itself and its treatments impose substantial physical and cognitive burdens on patients. Persistent symptoms such as fatigue, reduced muscle strength, and diminished exercise tolerance are frequently reported, limiting functional independence and daily activities [5,6,7,8]. In this regard, 36.7% to 54.6% of adults with cancer reported impairments in basic and instrumental activities of daily living [9]. Similarly, *“chemobrain”* or chemotherapy-related cognitive impairment (CRCI)—the neurocognitive side effects seen during or after cytotoxic cancer therapy—manifests as a significant decline in memory, attention, processing speed, and executive functions [10,11]. CRCI arises from the combined effects of oxidative stress, inflammation, and mitochondrial dysfunction caused by chemotherapy. These processes damage neurons, disrupt energy metabolism and synaptic communication, and impair brain repair mechanism. The resulting cognitive problems resemble accelerated brain aging and share biological pathways with neurodegenerative diseases such as Alzheimer’s [12]. Approximately, up to 50–80% of patients report noticing cognitive changes during treatment, and about one-quarter to one-third continue to have deficits years later [13,14]. Also, these cognitive symptoms have significant negative impacts on the quality of life of people with cancer [15,16]. Importantly, many of these adverse effects can endure even after treatment ends. A substantial minority of survivors (estimated 25–35%) continue to experience measurable cognitive deficits months or years following therapy [17,18].

Many daily activities demand simultaneous cognitive and motor effort—for instance, walking while talking on the phone. Such scenarios are studied in the dual-task (DT) paradigm where a person performs two independent tasks simultaneously [19]. One common measure is the dual-task cost (DTC), computed as the relative decline in performance under dual-task conditions versus single-task conditions [20]. In the cancer literature, although research is more limited than in aging or neurodegenerative disease, recent studies suggest that cancer survivors—especially those who have undergone chemotherapy and who have chemotherapy-induced neuropathy—exhibit impaired dual-task performance. In this regard, a previous study investigated the impact of chemotherapy on postural control in women with breast cancer, revealing significant impairments in balance, particularly in conditions involving sensory disturbances and dual tasks [21]. Similarly, some authors concluded that Timed Up and Go (TUG) tests, especially the cognitive version, are a reliable predictor of falls in breast cancer survivors [22]. Also, researchers found that a combination of chemotherapy-induced peripheral neuropathy (CIPN) and poor executive function in cancer survivors is associated with decreased gait stability during dual-task activities [23], suggesting that these combined impairments lead to a greater risk of instability. In addition, a randomized controlled trial in breast cancer survivors with CIPN showed that both social dance (adapted Tango) and home-based exercise improved dual-task performance, measured via Timed Up and Go with a cognitive task (counting backward), with improvements maintained at follow-up; this indicates both a baseline deficit and responsiveness to interventions under dual-task demands [24]. These findings suggest that the dual-task paradigm can reveal subtle deficits in motor–cognitive integration in cancer populations that may not be apparent in simpler single-task tests, making dual-task performance a sensitive marker of both cognitive and physical sequelae of treatment.

The existing literature has effectively established that cancer survivors face mobility and balance deficits, especially under dual-task conditions. Studies have focused on the negative effects of chemotherapy-induced neuropathy on gait stability [23] and the predictive value of mobility tests like the TUG for fall risk [22]. Furthermore, recent work shows that dual-task performance can be improved through interventions, confirming both a baseline deficit and a capacity for improvement in these patients [24]. However, there is a lack of research that directly compares functional strength and mobility under dual-task conditions across the full spectrum of the cancer journey, from active patients to long-term survivors, and against healthy controls. While previous studies have focused on gait and balance, there is a lack of studies investigating the role of strength, a crucial determinant of function in daily life [25].

Thus, the present study aims to quantify physical and cognitive function, including dual-task costs, in cancer survivors and patients on chemotherapy, compared to healthy controls. We will assess lower-body strength (30 s Chair Stand Test), functional mobility (Timed Up and Go test), and upper-body strength (30 s Arm Curl Test) in each participant under simple and dual-task conditions. The primary objectives are to compare (a) physical performance, (b) cognitive performance, and (c) dual-task cost across the groups. We hypothesize that cancer patients will show significantly poorer physical and cognitive function than controls, with correspondingly higher dual-task costs. Within the cancer cohort, we expect those undergoing active chemotherapy to have the greatest impairments, followed by the post-treatment survivors. These hypotheses stem from evidence that chemotherapy and related treatments exacerbate fatigue, muscle weakness, and cognitive fog [10,11,26]. By comparing active patients, survivors, and healthy individuals, our research will provide critical insights into how strength deficits manifest and evolve, and how they interact with cognitive demands at different stages of the cancer. This information is vital for developing targeted rehabilitation strategies that address the full range of physical and cognitive impairments in this population.

## 2. Materials and Methods

### 2.1. Participants

To define the sample size, the previous study by Morishita et al. [27], using the Timed Up and Go test as a reference, was used. We employed the G*Power software (version 3.1.9.7; Heinrich-Heine-Universität Düsseldorf, Düsseldorf, Germany; http://www.gpower.hhu.de/, accessed on 26 September 2025) to determine the minimum sample size. This software showed that 10 people per group were needed to achieve 90% power and a *p*-value < 0.05.

Finally, a total of 26 people with cancer and 18 healthy participants were recruited for this cross-sectional study. In addition, for subgroup analyses, the group of people with cancer was divided into two: (i) people who were currently undergoing chemotherapy (n = 11); and (ii) people who completed chemotherapy within the past 12 months (n = 15). The Spanish Association against Cancer (Cáceres, Spain) helped with the recruitment of people with cancer between April and June 2025.

Participants were eligible if they met the following inclusion criteria: (i) diagnosis of cancer and currently undergoing chemotherapy (treatment group) or completion of chemotherapy within the past 12 months (survivor group), or absence of cancer diagnosis (control group); (ii) age ≥ 18 years; (iii) ability to understand the study procedures and provide informed consent; and (iv) capacity to perform the physical fitness tests safely.

Exclusion criteria were as follows: (i) presence of severe musculoskeletal, neurological, or cardiovascular conditions limiting mobility or exercise performance; (ii) cognitive impairment preventing comprehension of instructions (MoCA score < 23); and (iii) any other medical condition deemed by the investigators to contraindicate participation.

All participants signed an informed consent where all the procedures and risks were explained. The research protocol was previously approved by the bioethics and biosafety committee of the University of Extremadura (ethics committee approval reference number: 49/2025) in accordance with the Declaration of Helsinki. Ethical approval for this cross-sectional study was obtained from the Bioethics and Biosafety Committee of the University of Extremadura (Ref. 49/2025). All procedures were conducted in accordance with the Declaration of Helsinki. Participant characteristics are detailed in Table 1.

### 2.2. Procedure

The assessment protocol began with the provision and explanation of informed consent, followed by the completion of a sociodemographic questionnaire, the International Physical Activity Questionnaire (IPAQ) [28], the Functional Assessment of Chronic Illness Therapy for quality of life (FACT-G) and fatigue (FACIT-F) [29,30,31], and a psychometric evaluation using the Montreal Cognitive Assessment (MoCA) [32]. Body composition was assessed using a bioelectrical impedance analyzer (BC-418 MA, TANITA, Tokyo, Japan). Subsequently, participants undertook a familiarization trial of the functional tests prior to their formal performance. All these tests were performed under both single-task and dual-task conditions. The cognitive task added to the dual-task condition consisted of subtracting by twos from a randomly selected odd number greater than 150. In addition, to minimize order effects, the sequence of the cognitive–motor tasks was randomized, alternating between single- and dual-task conditions. The same randomization procedure was applied to determine both the starting number and whether the baseline cognitive task was administered at the beginning or the end of the assessment.

All assessments were conducted in the Physical Condition and Quality of Life Laboratory at the Faculty of Sport Sciences, University of Extremadura. Environmental conditions were standardized throughout the testing sessions, with ambient temperature maintained between 20 and 24 °C and relative humidity controlled within a range of 40–60%.

### 2.3. Measurement of Outcomes

The Chair Stand Test (CST) evaluates lower body strength. Participants were instructed to rise to a full standing position and return to sitting as many times as possible within 30 s, with arms crossed over the chest. The total number of completed stands was recorded using the Chronopic contact platform (Chronojump, Barcelona, Spain).

The Arm Curl Test (ACT) assesses upper body strength. It is designed to measure functional physical capacity by recording the number of arm curls performed in 30 s while seated. In accordance with the SFT protocol, participants in this study used a 2.5 kg dumbbell (women) or a 4 kg dumbbell (men). Following the start signal, they were instructed to perform as many repetitions as possible within 30 s, aiming for maximal effort. The number of correctly executed repetitions was recorded as the test outcome.

The Timed Up and Go (TUG) assesses functional mobility and dynamic balance [33]. Participants began seated in a chair, stood up on the start signal, walked three meters at a comfortable pace, turned around, returned to the chair, and sat down. The time taken to complete the task was measured in seconds using the Chronopic contact platform (Chronojump, Barcelona, Spain).

The CST, ACT, and TUG belong to the Senior Fitness Test (SFT) battery [34]. This tool has been shown to predict treatment delay in older adults prior to chemotherapy and chemoradiation, supporting its utility as a practical tool for assessing functional fitness in oncology settings [35].

### 2.4. Statistical Analysis

Data were analyzed using jamovi software, version 2.6.26 [36]. All analyses were conducted after verifying the assumptions of normality and homogeneity of variance. Independent samples *t*-tests were applied to compare cancer patients with healthy controls. An exploratory analysis was conducted between patients undergoing treatment, cancer survivors, and healthy controls. Accordingly, a one-way analysis of variance (ANOVA) was conducted, followed by post hoc analyses using Tukey’s Honest Significant Difference test where applicable. Paired samples *t*-tests were conducted to compare performance between single-task and dual-task conditions. Dual-task cost (DTC) was calculated for all outcomes using the following formula:

%DTC = (Dual Task − Single Task)/Single Task × 100.(1)

A negative %DTC in repetitions, operations, and success indicates a decline in dual-task performance compared with the single-task condition, whereas positive values reflect an improvement. Conversely, for time and errors, which are inverse indicators of performance, positive %DTC values denote poorer dual-task performance, while negative values indicate better performance relative to the single task. Effect sizes were reported as Cohen’s *d* for *t*-tests and *η_p_*^2^ for ANOVA. Statistical significance was set at *p* < 0.05.

## 3. Results

### 3.1. Baseline Characteristics

Participant characteristics are shown in Table 1. No significant differences were observed between treatment, survivor, and control groups in age, weight, BMI, cognitive status, fatigue and quality of life, or physical activity (*p* > 0.05 for all). The majority of cancer diagnoses corresponded to breast cancer, and women predominated across groups.

### 3.2. Cancer Group vs. Control Group Comparison

Table 2 presents the intra- and intergroup comparisons between all cancer patients and healthy controls during single- and dual-task conditions.

In the CST, both groups completed fewer repetitions under dual-task than under single-task conditions (patients: *p* = 0.011, *d* = 0.65; controls: *p* = 0.001, *d* = 0.61). Controls performed more repetitions than patients in the single-task condition (*p* = 0.035, *d* = 0.37), but this difference was not significant during the dual-task one. For cognitive outcomes, correct operations and successful responses decreased significantly in controls (both *p* < 0.001).

In the ACT, controls showed a significant reduction in repetitions from single- to dual-task conditions (*p* < 0.001, *d* = 0.58). In the TUG, both groups required more time under the dual-task condition (patients: *p* < 0.001, *d* = 0.90; controls: *p* < 0.001, *d* = 0.95).

Table 3 shows the results of the DTC analyses between the cancer and control groups. In the ACT, patients exhibited lower motor costs (*p* = 0.046, *r* = 0.36) but higher cognitive (*p* = 0.005, *r* = 0.50) and success costs (*p* = 0.004, *r* = 0.52) compared with controls. No significant differences were observed in the CST and TUG tests.

### 3.3. Comparisons Between Cancer Patients Undergoing Chemotherapy, Cancer Survivors, and Healthy Control Individuals

Table 4 shows intra- and intergroup comparisons between cancer patients undergoing chemotherapy (treatment group), cancer survivors after chemotherapy, and healthy controls under single- and dual-task conditions.

In the CST, both survivors (*p* = 0.018, *r* = 0.82) and controls (*p* = 0.045, *r* = 0.57) performed significantly fewer repetitions under dual-task compared with single-task conditions. For cognitive outcomes, only controls showed significant reductions in correct operations (*p* = 0.003, *d* = 0.83) and successful responses (*p* = 0.003, *d* = 0.83) when dual-tasking. Survivors and the treatment group did not differ significantly within groups (*p*-value > 0.05).

In the ACT, controls showed a significant reduction in repetitions when dual-tasking (*p* < 0.001, *d* = 1.00). Controls also displayed significant decreases in correct operations (*p* = 0.003, *d* = 0.83) and successful responses (*p* = 0.002, *d* = 0.92).

For the TUG, all groups showed significantly longer times under dual-task compared with single-task conditions (treatment: *p* = 0.024, *r* = 0.76; survivors: *p* < 0.001, *r* = 0.97; controls: *p* < 0.001, *r* = 0.86). Regarding cognitive outcomes, only controls showed significant decreases in correct operations (*p* = 0.037, *r* = 0.66) and successful responses (*p* = 0.020, *d* = 0.60) during dual-tasking. Neither patients nor survivors displayed significant within-group differences.

Regarding between-group comparisons, no significant differences were observed in any of the physical fitness tests conducted (TUG, ACT, and CST).

Table 5 shows the results of the DTC analyses between treatment, survivors, and control groups. No significant between-group differences in DTC were observed for the CST or the TUG. In the ACT, significant differences were found for cognitive (*p* = 0.020, *ε*^2^= 0.18) and success costs (*p* = 0.015, *ε*^2^ = 0.19), with patients and survivors showing positive costs compared with negative values in controls.

## 4. Discussion

This study investigated physical and cognitive performance under single- and dual-task conditions in cancer patients undergoing chemotherapy, cancer survivors, and healthy controls. The main findings were that both cancer groups exhibited poorer functional strength and mobility compared with controls, particularly in the CST and TUG. Moreover, dual-task performance revealed additional impairments, with patients and survivors showing significantly higher dual-task costs in the ACT, especially for cognitive and success outcomes. These results suggest that cancer and its treatments negatively affect the integration of motor and cognitive function, and that dual-task testing is more sensitive than single-task assessments for detecting subtle functional deficits in this population.

Previous research has consistently shown that individuals with cancer or cancer survivors exhibit lower physical fitness compared to healthy controls when assessed through standardized functional tests. In this regard, survivors of ovarian cancer demonstrated significantly reduced performance in the six-minute walk test and peripheral muscle strength relative to healthy participants, reflecting impaired endurance and strength capacities [37]. Similarly, pediatric cancer survivors have been found to present lower cardiorespiratory fitness, muscle strength, and functional test performance—including sit-to-stand and handgrip strength—than age- and sex-matched controls [38,39]. Also, cancer survivors showed less hand and knee strength, balance, and mobility than healthy controls [27]. These findings are consistent with our results, where cancer patients tended to perform fewer repetitions in the ACT test under single-task conditions compared with controls. Taken together, the evidence indicates that reduced physical function is a pervasive consequence of cancer and its treatments, highlighting the importance of targeted rehabilitation strategies to restore strength and mobility in this population.

Also, our findings showed a dissociation in dual-task performance between cancer patients and healthy controls, which can suggest differences in attentional resource allocation [40]. While the TUG and CST revealed a decline in motor performance for both groups under dual-task conditions, task prioritization manifested uniquely in the ACT. Healthy controls showed a significant reduction in ACT repetitions, indicating a compromise between motor and cognitive tasks. In contrast, cancer patients did not show a significant decline in ACT performance, suggesting they expended attentional resources to maintain motor output, while their high cognitive DTC suggests they prioritized the cognitive component’s accuracy (as evidenced by positive DTC for success) at the expense of overall efficiency or a heightened cognitive load.

This pattern observed in cancer patients can be described as cognitive prioritization (or “posture-second”) strategy during complex tasks, concentrating more effort on the mental component at the expense of strength or speed. This is evidenced by positive dual-task costs (improvements) in cognitive success for patients, coupled with reduced motor output in the ACT. This strategy mirrors phenomena seen in other populations: limited attentional resources can be preferentially devoted to one task, improving performance there while worsening the other. In dual-task theory, this is known as a “posture second” approach, where individuals prioritize cognition over motor control [41,42]. Also, Blackwood and Rybicki [22] found TUG-Cognitive to be a reliable predictor of falls in breast cancer survivors. This highlights that while dual-tasking often serves to reveal latent deficits [21], in cancer populations it can also expose a strategic reallocation of attention, which is critical to consider when designing rehabilitation interventions aimed at improving both physical and cognitive function. Therefore, our findings are consistent with this model. By contrast, healthy controls showed the more balanced interference expected from capacity-sharing models, with both motor and cognitive performance declining under dual-task conditions.

These findings align with prior research on cancer survivors’ motor–cognitive integration. Reinmann, Bodmer, Koessler, Gligorov, and Bruyneel [21] found that women exposed to neurotoxic chemotherapy had impaired postural control, particularly under dual-task disturbances. Similarly, Monfort, Pan, Loprinzi, Lustberg, and Chaudhari [23] reported that survivors with CIPN and poorer executive function had reduced gait stability during challenging dual-task walking. Our data extend these insights by showing that even in simple strength and mobility tests, dual-task costs reveal vulnerabilities in cancer patients. In the TUG, both groups slowed under dual-tasking, but patients’ greater cognitive cost suggests they are more vulnerable when attentional resources must be divided. In this regard, a randomized controlled trial showed that integrative approaches may help mitigate side effects [43], potentially preventing the functional decline observed in our study. These patterns imply that treatment-related fatigue, muscle weakness, and cognitive impairment push survivors to reallocate effort during everyday activities, potentially impacting safety and independence. Therefore, as some studies investigating the practical clinical benefits of adjuvant treatments with non-pharmacological interventions have shown, such treatments could help to reduce fatigue-related to cancer treatments [44,45].

CRCI or *chemobrain* is characterized by difficulties in attention, processing speed, and executive functioning that can persist long after treatment [11,14]. The greater cognitive cost observed in our patients during the ACT under dual-task conditions may reflect precisely these deficits. When attentional resources are limited due to CRCI, patients may struggle to flexibly allocate cognitive effort across simultaneous demands, leading to disproportionate interference in complex motor tasks. In line with this, the tendency to prioritize cognitive accuracy in the ACT could be interpreted as a compensatory response to the perceived vulnerability of cognitive performance, even at the expense of motor efficiency. Thus, our findings provide behavioral evidence supporting the functional impact of CRCI, highlighting its role not only in isolated cognitive testing but also in ecologically valid dual-task scenarios that mirror daily life.

This study has some limitations that should be considered. The major limitation of this study was the absence of data on cancer stage and the specific chemotherapy regimens received by patients. Accordingly, future studies should stratify participants by disease severity and treatment type to better isolate the chemotherapy-specific effects on cognition and physical function. Secondly, the relatively small sample size may have limited the statistical power to detect subtle differences between groups, particularly in secondary outcomes. Another limitation of this study is the predominance of breast cancer cases and female participants, leading to an unbalanced distribution of cancer types and sexes. This may limit the generalizability of the results to other cancer types and to men. Future research should aim to include a more homogeneous or stratified sample by cancer type to better explore potential differences in outcomes, in addition to brief adjusted sensitivity analysis with this covariable. Another potential limitation of this study lies in the complexity of the cognitive task selected for the DT condition (serial subtractions by twos). While this dual-task approach proved sensitive in revealing deficits where single-task assessments failed, the specific cognitive load applied may have been insufficiently demanding to maximize the observed interference between groups. Prior studies in cancer patients that reported significant DT costs utilized more challenging tasks, such as subtraction by 3 [22,24] and subtraction by 7 [23]. However, it is crucial to consider the complexity of task selection in light of emerging research. For example, Bianchini et al. [46] found that the DTC decreased with increasing cognitive task complexity in a cohort spanning various neurological conditions and age groups. Specifically, they noted that the simplest cognitive task yielded the largest and most stable DTC, while the most complex task resulted in a lower or even negative DTC. Applying this finding to our study, while a more challenging task (like subtraction by 7) might have been anticipated to maximize the difference, it could have paradoxically led to a reduced or inconsistent cognitive DTC due to the participants’ clear cognitive prioritization strategy observed in our results. Our finding that patients did not show a significant decline in ACT performance suggests they already prioritized the motor task, and a highly complex cognitive task might have been either fully deprioritized or, conversely, facilitated by the simultaneous motor activity (cross-talk), thereby masking the true interference. Therefore, the simple nature of the serial subtractions by twos may have been better suited to detecting a robust positive cognitive DTC in this specific population. This highlights the inherent difficulty in selecting the optimal cognitive load and suggests that future studies should explore a spectrum of complexity to fully characterize the effects of cancer and its treatments on motor–cognitive integration.

The observed results have several practical applications. First, clinicians should be aware that standard single-task assessments may underestimate patients’ real-world impairments. Incorporating dual-task evaluations (e.g., TUG with cognitive load) can unmask hidden deficits in motor–cognitive integration that are not apparent in simple tasks. Second, rehabilitation programs should address both physical and cognitive domains together. The fact that patients responded positively to dual-task-focused interventions (such as adapted dance or home exercise) [24] shows that training can improve dual-task function. Interventions combining strength training, balance exercises, and cognitive tasks (dual-task training) may yield the greatest benefits. Finally, survivors and caregivers should be informed about potential cognitive side effects and experience of CRCI [47], and trained in strategies (like prioritization and compensatory techniques) to manage everyday multitasking safely.

## 5. Conclusions

Cancer patients and survivors showed greater impairments in strength and mobility under dual-task conditions, with higher cognitive costs supporting the impact of CRCI. Dual-task testing appears to be more sensitive than single-task assessments and should be considered in rehabilitation to address both physical and cognitive deficits. Further research is warranted in this field such as employing larger sample sizes, longitudinal studies to track progression, and stratified analyses by specific cancer types, and including a spectrum of complexity in the cognitive task to characterize the effects of cancer and its treatment on motor–cognitive integration.

## Figures and Tables

**Table 1:** Demographic, clinical, and functional characteristics of the study sample.

Variables		Treatment Mean (SD)	Survivors Mean (SD)	Controls Mean (SD)	*p*-Value
Age (years)		49.82 (6.57)	54.80 (9.84)	51.06 (7.02)	0.246
Weight (kg)		64.81 (12.74)	66.81 (11.82)	69.24 (14.21)	0.674
BMI (kg/m^2^)		24.40 (4.87)	25.50 (3.86)	25.06 (4.44)	0.818
MoCA		27.36 (2.11)	27.67 (1.99)	27.56 (1.58)	0.919
FACIT-F total score		121.45 (19.88)	122.93 (19.17)	115.22 (15.01)	0.427
IPAQ (n)	Active	3	9	7	0.455
	Moderate	7	4	7	
	Sedentary	1	2	4	
Cancer type (n)	Breast	8	7	-	-
	Appendix	1	1	-	
	Colorectal	0	2	-	
	Retroperitoneal	1	0	-	
	Multiple myeloma	0	1	-	
	Testicle	1	0	-	
	Hemat lymphoma	0	1	-	
	Pancreas	0	1	-	
	Cervix	1	1	-	
Sex (n)	Female	9	12	7	0.370
	Male	2	3	7	

SD: standard deviation; BMI: body mass index; MoCA: Montreal Cognitive Assessment; FACIT-F: Functional Assessment of Chronic Illness Therapy for fatigue; IPAQ: International Physical Activity Questionnaire.

**Table 2:** Results of the comparisons within and between cancer patients (n = 26) and healthy individuals (n = 18).

	Variables	Group	Single-Task	Dual-Task	Within-Group Comparison		Between-Group Comparisons			
			Mean (SD)	Mean (SD)	*p*-Value	ES	Single-Task		Dual-Task	
							*p*-Value	ES	*p*-Value	ES
CST	Repetitions	Cancer	13.00 (3.79)	11.69 (2.87)	0.011 * ^b^	0.65	0.035 * ^e^	0.37	0.142 ^c^	0.46
		Control	14.06 (1.83)	12.94 (2.51)	0.001 * ^b^	0.61				
	Operations	Cancer	17.77 (6.37)	15.50 (5.31)	0.054 ^a^	0.4	0.227 ^c^	0.38	0.936 ^d^	0.02
		Control	20.06 (5.63)	15.61 (3.81)	<0.001 * ^a^	0.56				
	Success	Cancer	17.65 (6.52)	15.12 (5.29)	0.029 * ^a^	0.45	0.285 ^c^	0.33	0.908 ^d^	0.03
		Control	19.78 (6.22)	15.28 (3.94)	<0.001 * ^a^	0.6				
	Errors	Cancer	0.12 (0.33)	0.38 (0.70)	0.097 ^b^	0.67	0.948 ^e^	0.01	0.717 ^e^	0.05
		Control	0.28 (0.83)	0.33 (0.77)	0.122 ^b^	0.5				
ACT	Repetitions	Cancer	17.92 (4.99)	16.65 (5.00)	0.110 ^a^	0.33	0.055 ^c^	0.62	0.970 ^c^	0.01
		Control	20.88 (4.50)	16.71 (3.10)	<0.001 * ^a^	0.58				
	Operations	Cancer	17.77 (6.37)	17.77 (5.04)	1.000 ^a^	0	0.227 ^c^	0.38	0.742 ^c^	0.1
		Control	20.06 (5.63)	17.29 (3.80)	0.055 ^a^	0.3				
	Success	Cancer	17.65 (6.52)	17.65 (5.09)	1.000 ^a^	0	0.285 ^c^	0.33	0.661 ^c^	0.14
		Control	19.78 (6.22)	17.00 (4.15)	0.046 * ^a^	0.31				
	Errors	Cancer	0.12 (0.33)	0.12 (0.33)	1.000 ^b^	0	0.948 ^e^	0.01	0.893 ^e^	0.02
		Control	0.28 (0.83)	0.29 (0.85)	0.407 ^b^	0.33				
TUG	Time	Cancer	6.12 (0.89)	7.14 (1.42)	<0.001 * ^b^	0.9	0.692 ^c^	0.12	0.567 ^e^	0.1
		Control	6.02 (0.84)	6.84 (1.42)	<0.001 * ^b^	0.95				
	Operations	Cancer	4.42 (2.06)	4.35 (1.60)	0.809 ^b^	0.07	0.309 ^e^	0.18	0.286 ^c^	0.33
		Control	4.89 (2.19)	3.83 (1.47)	0.118 ^b^	0.32				
	Success	Cancer	4.35 (2.19)	4.15 (1.78)	0.670 ^b^	0.12	0.535 ^c^	0.19	0.253 ^e^	0.2
		Control	4.78 (2.34)	3.67 (1.41)	0.083 ^b^	0.35				
	Errors	Cancer	0.08 (0.39)	0.19 (0.49)	0.490 ^b^	0.4	0.397 ^e^	0.07	0.970 ^e^	0.01
		Control	0.11 (0.32)	0.17 (0.38)	0.340 ^b^	0.39				

^a^ Student’s *t* paired samples test and Cohen’s *d* effect size; ^b^ Wilcoxon *W* test and rank biserial correlation effect size; ^c^ Student’s *t* independent samples test and Cohen’s *d* effect size; ^d^ Welch’s *t* test and Cohen’s *d* effect size; ^e^ Mann–Whitney *U* test and rank biserial correlation effect size; SD: standard deviation; ES: effect size; CST: Chair Stand Test; ACT: Arm Curl Test; TUG: Timed Up and Go; * *p *< 0.05.

**Table 3:** Results of the dual-task cost comparisons between cancer patients (n = 26) and healthy participants (n = 18).

Test	Variables	Cancer Mean (SD)	Control Mean (SD)	*p*-Value	ES
Chair Stand Test	DTC motor (%) ^a^	−8.07 (16.39)	−7.66 (15.68)	0.934 ^a^	0.03
	DTC cognitive (%) ^b^	−3.77 (41.69)	−16.81 (27.46)	0.504 ^b^	0.12
	DTC success (%) ^b^	−3.24 (51.18)	−15.14 (31.26)	0.633 ^b^	0.09
Arm Curl Test	DTC motor (%) ^b^	−2.83 (34.03)	−16.59 (17.39)	0.046 * ^b^	0.36
	DTC cognitive (%) ^b^	7.54 (34.44)	−18.02 (0.31)	0.005 * ^b^	0.50
	DTC success (%) ^b^	9.40 (37.91)	−18.89 30.37)	0.004 * ^b^	0.52
Timed Up and Go	DTC motor (%) ^b^	16.75 (16.30)	13.63 (15.92)	0.500 ^b^	0.12
	DTC cognitive (%) ^b^	11.27 (50.61)	−5.24 (82.61)	0.088 ^b^	0.30
	DTC success (%) ^b^	0.95 (53.63)	−6.81 (82.90)	0.171 ^b^	0.24

^a^ Student’s *t* test and Cohen’s *d*; ^b^ Mann–Whitney’s *U* test and rank biserial correlation; SD: standard deviation; ES: effect size; DTC: dual-task cost. * *p *< 0.05.

**Table 4:** Results of the comparisons within and between cancer patients undergoing chemotherapy, cancer survivors after chemotherapy and healthy individuals.

Test	Variables	Group	Single-Task	Dual-Task	Within-Group Comparison		Between-Group Comparisons			
			Mean (SD)	Mean (SD)	*p*-Value	ES	Single-Task		Dual-Task	
							*p*-Value	ES	*p*-Value	ES
CST	Repetitions	Treatment	13.18 (3.28)	12.18 (2.68)	0.283 ^b^	0.42	0.069 ^e^	0.12	0.255 ^c^	0.06
		Survivors	12.87 (4.24)	11.33 (3.04)	0.018 * ^b^	0.82				
		Control	14.06 (1.83)	12.94 (2.51)	0.045 * ^b^	0.57				
	Operations	Treatment	18.18 (4.40)	16.64 (3.98)	0.379 ^a^	0.28	0.466 ^c^	0.04	0.609 ^d^	0.03
		Survivors	17.47 (7.64)	14.67 (6.10)	0.090 ^a^	0.47				
		Control	20.06 (5.63)	15.61 (3.81)	0.003 * ^a^	0.83				
	Success	Treatment	18.00 (4.65)	16.09 (4.01)	0.268 ^a^	0.35	0.554 ^c^	0.03	0.700 ^d^	0.02
		Survivors	17.40 (7.77)	14.40 (6.10)	0.068 ^a^	0.51				
		Control	19.78 (6.22)	15.28 (3.94)	0.003 * ^a^	0.83				
	Errors	Treatment	0.18 (0.40)	0.55 (0.82)	0.265 ^b^	0.7	0.688 ^e^	0.02	0.567 ^e^	0.03
		Survivors	0.07 (0.26)	0.27 (0.59)	0.345 ^b^	0.6				
		Control	0.28 (0.83)	0.33 (0.77)	0.850 ^b^	0.2				
ACT	Repetitions	Treatment	18.55 (5.94)	17.45 (3.98)	0.383 ^a^	0.28	0.139 ^c^	0.09	0.728 ^c^	0.02
		Survivors	17.47 (4.32)	16.07 (5.70)	0.196 ^a^	0.35				
		Control	20.88 (4.50)	16.71 (3.10)	<0.001 * ^a^	1				
	Operations	Treatment	18.18 (4.40)	18.45 (2.50)	0.855 ^a^	0.06	0.466 ^c^	0.04	0.770 ^c^	0.01
		Survivors	17.47 (7.64)	17.27 (6.34)	0.847 ^a^	0.05				
		Control	20.06 (5.63)	17.29 (3.80)	0.003 * ^a^	0.83				
	Success	Treatment	18.00 (4.65)	18.27 (2.57)	0.862 ^a^	0.05	0.554 ^c^	0.03	0.776 ^c^	0.01
		Survivors	17.40 (7.77)	17.20 (6.41)	0.849 ^a^	0.05				
		Control	19.78 (6.22)	17.00 (4.15)	0.002 * ^a^	0.92				
	Errors	Treatment	0.18 (0.40)	0.18 (0.40)	1.000 ^b^	0	0.688 ^e^	0.02	0.688 ^e^	0.02
		Survivors	0.07 (0.26)	0.07 (0.26)	1.000 ^b^	0				
		Control	0.28 (0.83)	0.29 (0.85)	0.371 ^b^	1				
TUG	Time	Treatment	5.90 (0.55)	6.76 (1.10)	0.024 * ^b^	0.76	0.517 ^d^	0.03	0.480 ^e^	0.03
		Survivors	6.28 (1.06)	7.42 (1.59)	<0.001 * ^b^	0.97				
		Control	6.02 (0.84)	6.84 (1.42)	<0.001 * ^b^	0.86				
	Operations	Treatment	4.09 (1.76)	4.45 (1.21)	0.609 ^b^	0.25	0.493 ^e^	0.03	0.544 ^c^	0.03
		Survivors	4.67 (2.29)	4.27 (1.87)	0.502 ^b^	0.24				
		Control	4.89 (2.19)	3.83 (1.47)	0.037 * ^b^	0.66				
	Success	Treatment	3.91 (2.07)	4.45 (1.21)	0.412 ^a^	0.26	0.524 ^e^	0.03	0.465 ^c^	0.04
		Survivors	4.67 (2.29)	3.93 (2.12)	0.257 ^a^	0.31				
		Control	4.78 (2.34)	3.67 (1.41)	0.020 * ^a^	0.6				
	Errors	Treatment	0.18 (0.60)	0.00 (0.00)	1.000 ^b^	1	0.439 ^e^	0.04	0.183 ^e^	0.08
		Survivors	0.00 (0.00)	0.33 (0.62)	0.089 ^b^	1				
		Control	0.11 (0.32)	0.17 (0.38)	0.773 ^b^	0.33				

^a^ Student’s *t* paired samples test and Cohen’s *d* effect size; ^b^ Wilcoxon *W* test and rank biserial correlation effect size; ^c^ Fisher’s test and *η_p_*^2^ effect size; ^d^ Welch’s test and *η_p_*^2^ effect size; ^e^ Kruskal–Wallis *H* test and *ε*^2^ effect size; SD: standard deviation; ES: effect size; CST: Chair Stand Test; ACT: Arm Curl Test; TUG: Timed Up and Go; * *p *< 0.05.

**Table 5:** Results of the dual-task cost comparisons between cancer patients undergoing chemotherapy, cancer survivors, and healthy participants.

Test	Variables	Treatment Mean (SD)	Survivors Mean (SD)	Control Mean (SD)	*p*-Value	ES
CST	DTC motor (%) ^a^	−5.67 (19.22)	−9.83 (14.42)	−7.66 (15.68)	0.934 ^a^	0.03
	DTC cognitive (%) ^b^	−4.80 (28.18)	−3.02 (50.34)	−16.81 (27.46)	0.504 ^b^	0.12
	DTC success (%) ^b^	−6.92 (27.15)	−0.54 (64.29)	−15.14 (31.26)	0.633 ^b^	0.09
ACT	DTC motor (%) ^b^	3.44 (44.97)	−7.43 (23.88)	−16.59 (17.39)	0.046 * ^b^	0.36
	DTC cognitive (%) ^b^	6.93 (29.60)	7.99 (38.63)	−18.02 (30.31)	0.005 * ^b^	0.50
	DTC success (%) ^b^	8.25 (34.11)	10.25 (41.63)	−18.89 (30.37)	0.004 * ^b^	0.52
TUG	DTC motor (%) ^b^	14.89 (18.33)	18.12 (15.16)	13.63 (15.92)	0.500 ^b^	0.12
	DTC cognitive (%) ^b^	24.02 (53.55)	1.92 (48.00)	−5.24 (82.61)	0.088 ^b^	0.30
	DTC success (%) ^b^	14.92 (47.51)	−9.30 (57.07)	−6.81 (82.90)	0.171 ^b^	0.24

^a^ Fisher’s test and *η_p_*^2^; ^b^ Kruskal–Wallis *H* test and *ε*^2^; SD: standard deviation; ES: effect size; DTC: dual-task cost; * *p* < 0.05.

## Data Availability

The data presented in this study are available on reasonable request from the corresponding author. The data are not publicly available due to privacy and ethical restrictions.

## Abbreviations

The following abbreviations are used in this manuscript:

CRCI	Chemotherapy-related cognitive impairment
DT	Dual task
DTC	Dual-task cost
TUG	Timed Up and Go
CIPN	Chemotherapy-induced peripheral neuropathy
IPAQ	International Physical Activity Questionnaire
FACT-G	Functional Assessment of Chronic Illness Therapy for quality of life
FACIT-F	Functional Assessment of Chronic Illness Therapy for fatigue
MOCA	Montreal Cognitive Assessment
CST	Chair Stand Test
ACT	Arm Curl Test
SFT	Senior Fitness Test
BMI	Body mass index
